# Eradication of Myrosinase-Tethered Cancer Cells by Allyl Isothiocyanate Derived from Enzymatic Hydrolysis of Sinigrin

**DOI:** 10.3390/pharmaceutics14010144

**Published:** 2022-01-07

**Authors:** Ammar Tarar, Esmael M. Alyami, Ching-An Peng

**Affiliations:** Department of Chemical & Biological Engineering, University of Idaho, Moscow, ID 83844, USA; tara9039@vandals.uidaho.edu (A.T.); alya7030@vandals.uidaho.edu (E.M.A.)

**Keywords:** sinigrin, myrosinase, allyl isothiocyanate, phytochemicals, core streptavidin, adenocarcinoma A549 lung cancer cell, cancer therapy, prodrug

## Abstract

Sinigrin is present in significant amounts in cruciferous vegetables. Epidemiological studies suggest that the consumption of such vegetables decreases the risk of cancer, and the effect is attributed mainly to allyl isothiocyanate (AITC), a hydrolysis product of sinigrin catalyzed by myrosinase. Anticancer activity of AITC has been previously investigated for several cancer models, but less attention was paid to delivering AITC on the target site. In this study, the gene sequences of core streptavidin (coreSA) and myrosinase (MYR) were cloned in a pET-30a(+) plasmid and transformed into BL21(DE3) *E. coli* competent cells. The MYR-coreSA chimeric protein was expressed and purified using immobilized metal affinity chromatography and further characterized by gel electrophoresis, Western blot, and enzyme activity assay. The purified MYR-coreSA chimeric protein was tethered on the outer membrane of biotinylated adenocarcinoma A549 cells and then treated with various concentrations of sinigrin. Our results showed that 20 µM of sinigrin inhibited the growth of A549 cells tethered with myrosinase by ~60% in 48 h. Furthermore, the levels of treated cells undertaken apoptosis were determined by Caspase-3/7 activation and Annexin-V. In summary, sinigrin harnessed like a prodrug catalyzed by myrosinase to the production of AITC, which induced cell apoptosis and arrested the growth of lung cancer cells.

## 1. Introduction

Cancer is a serious health concern and the leading cause of death throughout the world. Recent epidemiologic studies show that utilization of cruciferous foods can reduce the event of a threat and diminish the frequency of malignant growth [1,2]. For instance, the intake of cruciferous vegetables helps to reduce the risk of ovarian [3] and colon cancer [4,5], lung cancer [6], and prostate cancer [7,8]. Cruciferous vegetables are in the *Brassicaceae* family that includes broccoli, cabbage, wasabi, mustard cauliflower, horseradish, etc. Allyl isothiocyanate (AITC) is an organosulfur phytochemical found in the sources of cruciferous vegetables [9]. The pungent flavor of these vegetables is mainly because of AITC, which is also a chemopreventive agent. It has fungicidal and bactericidal activities against a plethora of microorganisms, and its anticancer activities were demonstrated in different cancer cell lines in vitro [10] and some animal tumor models in vivo [11]. Quite a few studies demonstrated that AITC elicits cell apoptosis and causes cell cycle arrest through modulation of various genes involved in proliferation and survival in malignant cell lines such as cervical cancer [10], colorectal carcinoma [9,12,13], breast adenocarcinoma [14], hepatoma [15], prostate cancer [11], bladder malignancy [16,17,18], brain glioma [19], and oral cancer [20].

**Figure 1:**
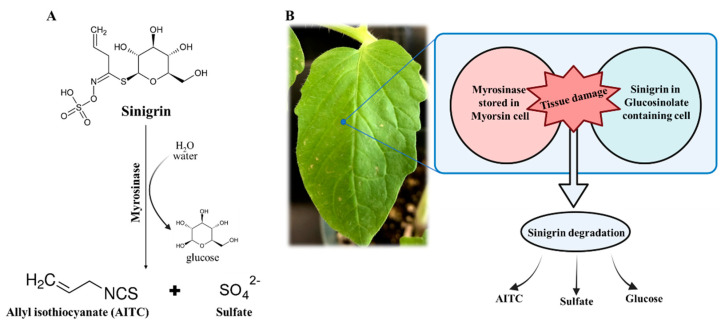
(**A**) Hydrolysis reaction of sinigrin catalyzed through myrosinase to produce AITC; (**B**) schematic illustration of plant tissue damage and ensuing interaction of sinigrin and myrosinase with AITC production.

AITC is stored in plants as its precursor called sinigrin (*a.k.a*. allyl glucosinolate). Sinigrin and its hydrolytic myrosinase are stored in physically separated compartments of the *Brassicaceae* family to prevent self-intoxication [21] in the intact plant tissue. Upon tissue disruption, hydrolytic myrosinase enzyme comes in contact with sinigrin, and then sinigrin is hydrolyzed by myrosinase to rapidly release lipophilic, irritant, and toxic AITC [22] (as illustrated in Figure 1). In this study, we developed a sinigrin–myrosinase system to eradicate adenocarcinoma A549 lung cancer cells. A fusion protein composed of myrosinase and core streptavidin (coreSA) was obtained through bacterial expression and purifying using immobilized metal affinity chromatography and then tethered on the outer membrane of biotinylated A549 cancer cells. After treating with sinigrin, the myrosinase decorated on A549 cells produced AITC, which induced cell apoptosis.

## 2. Materials and Methods

### 2.1. Materials

Lysogeny broth (LB) media, isopropyl-β-D-thiogalactopyranoside (IPTG), penicillin–streptomycin, Annexin-V-Flous Staining kit, 2-mercaptoethanol (ßME), and imidazole were purchased from Sigma-Aldrich (St. Louis, MO, USA). Dulbecco’s modified Eagle’s medium (DMEM) culture media, fetal bovine serum (FBS), 0.25% trypsin-EDTA, bicinchoninic acid (BCA) protein assay kit, bovine serum albumin (BSA) standard, bacterial protein extraction reagent (B-PER), streptavidin PE-Cy5.5, biotin-X DHPE (N-((6-(biotinoyl)amino)hexanoyl)-1,2-dihexadecanoyl-sn-glycero-3-phosphoethanolamine, triethylammonium salt), protease inhibitor, B-PER lysing agent, protein concentrator PES (MWCO = 50 K), HisPur cobalt-NTA resin, enhanced chemiluminescence (ECL), EDTA-free protease inhibitor, phosphate-buffered saline (PBS), streptavidin monoclonal antibody, and CellEvent™ Caspase-3/7 Green Detection Reagent were all purchased from Thermo Fisher Scientific (Waltham, MA, USA). Dimethyl sulfoxide (DMSO) and kanamycin sulfate were obtained from Santa Cruz Biotech (Dallas, TX, USA). Adenocarcinoma A549 lung cancer cell line was obtained from the ATCC (Manassas, VA, USA). High-fidelity Phusion polymerase, Monarch PCR & DNA cleanup kit, T7 Express BL21(DE3)-competent *E. coli*, restriction enzymes (EcoRV, XhoI, BamHI, and EcoRI), T4 DNA ligase, Blunt/TA Ligase Master Mix, and NEB5α-competent *E. coli* (subcloning efficiency) were all obtained from New England BioLabs (NEB) (Ipswich, MA, USA). Laemmli sample buffer, 0.2 µm nitrocellulose membrane, Tris/glycine/SDS buffer, and Tris-buffer saline with 0.1% Tween-20 (TBST) were purchased from Bio-Rad (Hercules, CA, USA). IgG horseradish peroxidase (HRP)-conjugated antibody was obtained from R&D Systems (Minneapolis, MN, USA). His-tag monoclonal antibody was purchased from GenScript (Piscataway, NJ, USA). Plasmid pET-30a(+) was acquired from Addgene (Watertown, MA). Plasmid Miniprep kit was obtained from Qiagen (Germantown, MD, USA). Rapid Coomassie blue stain was purchased from Research Products International (Mount Prospect, IL, USA). Primers listed in Table 1 were all purchased from Integrated DNA Technologies (Coralville, IA, USA).

### 2.2. Construction of Myrosinase-Core Streptavidin (MYR-coreSA) Encoding Plasmid

**Figure 2:**
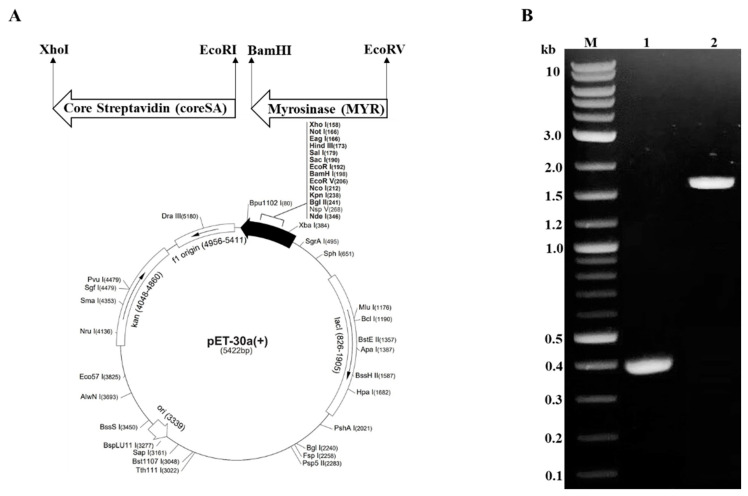
Construction of pMT002 vector. (**A**) Vector map shows the cloning site of MYR between EcoRV/BamHI and coreSA between EcoRI /XhoI, respectively. The direction of arrows shows the 5′ to 3′ end of each insert; (**B**) DNA agarose gel confirmation of PCR products (M: DNA ladder, Lane 1: coreSA, ~0.4 kb, Lane 2: MYR, ~1.6 kb) cloned from pMT002.

The myrosinase cDNA from the *Arabidopsis thaliana* (TGG1) gene was cloned from the plasmid encoding TGG1, acquired from DNASU Plasmid Repository of Arizona State University (Plasmid ID No: AtCD00804829; Tempe, AZ, USA), using the primers listed in Table 1 for the polymerase chain reaction (PCR). The PCR reaction for MYR was performed using Phusion DNA polymerase (NEB) in a thermocycler (Bio-Rad, Hercules, CA, USA). The operation setting was as follows: initial denaturation at for 30 s at 98 °C, followed by 30 cycles of denaturation at 98 °C for 10 s, annealing for 30 s at 66 °C, and extension for 15 s at 72 °C, and final extension for 5 min at 72 °C. The PCR reaction for the coreSA gene sequence was performed using pSTE2-215 (yol) plasmid [23] as template. The primers used for PCR are given in Table 1. CoreSA PCR reactions were performed with the aforementioned DNA polymerase, times, temperatures, and cycles, except for the annealing temperature of 62 °C. Both PCR products were analyzed by running on 1% of agarose gel electrophoresis. Plasmid vector pET-30a(+) and insert (coreSA PCR product) were cut with restriction enzymes EcoRI and XhoI. The digested vector and insert were ligated by T4 DNA ligase to get the pET-30a(+)-coreSA (*a.k.a* pMT001) plasmid, which was transformed to subcloning efficiency NEB5α-competent cells and purified by plasmid miniprep kit (Qiagen). Next, the pMT001 plasmid and insert (MYR PCR product) were digested with restriction enzymes EcoRV and BamHI and subsequently ligated with Blunt/TA Ligase Master Mix to get the pET30a-coreSA-MYR (*a.k.a* pMT002) plasmid. The ligation product (pMT002 plasmid) was transformed with subcloning efficiency NEB5α-competent cells and purified by plasmid miniprep kit (Qiagen). As shown in the vector map of Figure 2A, The pET30a-coreSA-MYR plasmid (i.e., pMT002) was constructed by inserting the gene sequences of coreSA and MYR in between EcoRI/XhoI and EcoRV/BamHI of pET-30a(+), respectively.

### 2.3. Expression and Purification of MYR-coreSA Recombinant Protein

The constructed pMT002 plasmid was transformed into T7 Express BL21(DE3)-competent *E. coli* cells cultured in LB media at 37 °C and 225 rpm. When the optical density (OD) at 600 nm reached 0.6, the IPTG was added to a final concentration of 0.4 mM and kept shaking overnight at 22 °C and 225 rpm. Cells were then harvested by centrifugation at 4500× *g* for 15 min. The cell pellet (~1 g) was resuspended in 5 mL of lysis buffer (B-PER supplemented with 50 mM Tris-HCl, pH 7.5) and incubated for 10 m at room temperature to extract bacterial protein. To preserve protein stability, EDTA-free protease inhibitor (Thermo Fisher Scientific) was also added to the lysis buffer. To maximize the protein extraction efficiency, the cell pellet with the added lysing buffer was then sonicated 15 times (10 s pulse with 10 s rest in between) with power output set at seven (Misonix, Farmingdale, NY, USA). Finally, the lysate was centrifuged for 15 min at 18,000× *g* under 4 °C. The collected supernatant consisting of crude protein was preserved at −20 °C.

The MYR-coreSA fusion protein was purified from crude protein using cobalt-NTA affinity chromatography. The crude protein and binding buffer (10 mM imidazole in 1× PBS, pH = 7.4) were mixed (1:1 ratio) and gently shaken in HisPur cobalt-NTA resin 4 °C for 1 h. The mixture was loaded onto the column and flow-through was collected, then washed with 5 resin bed volumes of wash buffer (10 mM imidazole in 1× PBS, pH = 7.4) and eluted with 3 resin bed volumes of elution buffer (250 mM imidazole in 1× PBS, pH = 7.4). The elutions were concentrated using protein concentrator PES (MWCO = 50 K). The optical density (OD) at 280 nm for all the washes and elutions was measured by a microplate reader (SpectraMax M2e; Molecular Devices, Sunnyvale, CA, USA).

### 2.4. SDS-PAGE, Western Blot, and BCA Assay

Eluted MYR-coreSA and the crude protein were diluted (1:2 ratio) with 2× Laemmli sample buffer (Bio-Rad) supplemented with 5% βME and heated to 80 °C for 10 min. The SDS-PAGE was performed in 12% polyacrylamide gels at 200 V for 40 min using 1× Tris/glycine/SDS buffer (Bio-Rad) and the gel was stained with a rapid Coomassie blue stain (Research Products International). For Western blot analysis, eluted MYR-coreSA was run on an SDS-PAGE and then transferred to 0.2 µm nitrocellulose membranes (Bio-Rad) using the semi-dry system (Bio-Rad) for 1 h at 20 V. The membranes were blocked for 1 h at room temperature with 5% BSA in Tris-buffered saline with 0.1% Tween-20 (TBST). After that, the membranes were washed thrice gently with TBST and incubated with two different primary antibodies in blocking buffer (1:1000 dilution of streptavidin monoclonal antibody and His-tag monoclonal antibody, respectively) overnight at 4 °C. The membranes were rinsed again thrice with TBST and allowed to react for 1 h with secondary antibody in blocking buffer (1:1000 dilution of mouse IgG HRP-conjugated antibody) at room temperature. The coreSA-MYR fusion protein was detected by horseradish peroxidase (HRP) activity using ECL substrate (Thermo Fisher Scientific) imaged by chemiluminescence imager (PXi Syngene, Frederick, MD, USA).

The BCA protein assay kit (Thermo Fisher Scientific) was used according to the manufacturer’s provided protocols. Briefly, the BSA standard (2000 µg/mL) was diluted (25, 125, 250, 500, 750, 1000, and 1500 µg/mL) in 1× PBS. Mix 50 parts of BCA reagent A and 1 part of BCA reagent B to prepare the working reagent solution (WR). Then, 200 µL of WR was mixed with 30 µL of each elution protein and standard and incubated at 37 °C for 30 min. The absorbance at 562 nm was measured using a SpectraMax M2e microplate reader (Molecular Devices). The absorbance of the BSA standard was used to plot the BSA standard curve for quantifying elution protein through its absorbance.

### 2.5. Surface Biotinylation and MYR-coreSA Tethering on A549 Cancer Cells

A549 cells were cultivated in a 6-well plate containing DMEM culture medium that is supplemented with 1% penicillin–streptomycin and 10% FBS under 5% CO_2_ with balanced humidifier air and 37 °C. The next day, when the cells attached and spread on the culture surface, the conditioned culture medium was replaced with fresh media containing 0.02 mg/mL biotin-X DHPE and incubated for an additional 24 h. To confirm biotinylation, the media were discarded, and cells were incubated for 1 h in streptavidin PE-Cy5.5 to bind the streptavidin with the biotinylated cell surface. Then, the cells were gently washed with PBS to discard any unbounded streptavidin PE-Cy5.5. Phase-contrast along with fluorescence images of cells were taken with a Leica DMi8 microscope equipped with a Leica EC3 camera (Leica Microsystems, Wetzlar, Germany). For tethering MYR-coreSA fusion protein on the surface of A549 cells, the biotinylated media were discarded, and the fusion protein (2 µg) was incubated for 1 h, followed by a gentle wash with 1× PBS to discard unbounded fusion protein.

### 2.6. Enzyme Activity Assay

The enzyme activity of recombinant MYR-coreSA protein was measured using a microplate reader (SpectraMax M2e) according to the protocols described previously with slight modification [24]. In brief, the stock solution of sinigrin and myrosinase was prepared separately in a 50 mM sodium citrate buffer (pH 7). A variety of concentrations of sinigrin (0, 6, 9, 12, 18, 25, 37, 50, 100, 150, 200 µM) were set up at 37 °C. Each reaction was initiated with the addition of myrosinase (100 ng) and terminated after 1, 3, 5, 7, and 10 min by heating each reaction at 95 °C for 5 min. Glucose produced in each reaction at a specific time was measured using a glucose assay kit according to the manufacturer’s instruction protocols. The velocity of reaction with respect to each concentration of sinigrin was plotted, and the Hanes plot was used to find the *K_m_* and *V_max_*.

### 2.7. Cytotoxicity Assay

A549 cells were cultured in a 24-well plate (Thermo Fisher Scientific) and tethered with MYR-coreSA as described earlier. Various concentrations (0, 0.625, 1.25, 2.5, 5, 10, 15, and 20 µM) of sinigrin were added and viability after 48 h was quantified using MTT assay. The A549 cells were directly treated with the same concentrations of AITC dissolved in DMSO. The phase-contrast images were also taken after 48 h with a Leica DMi8 microscope equipped with a Leica EC3 camera (Leica Microsystems) to observe the cell morphological changes.

### 2.8. Apoptosis Assay

The Annexin-V-Flous staining kit was used along with propidium iodide (PI) as per manufacturer protocols. Briefly, 20 µL of Annexin-V labeling reagent and 20 µL of PI solution was diluted in 1 mL of incubation buffer to make a working solution. Then, 10^6^ cells were incubated in 0.1 mL of working solution for 20 min at room temperature followed by flow cytometric analysis (Accuri C6; BD Biosciences, San Jose, CA, USA). For Caspase-3/7 detection, 10^6^ cells were incubated for 30 min in Caspase-3/7 green detection agent with a final concentration of 2 µM, and images were taken under a fluorescent microscope (Leica DMi8 microscope).

### 2.9. Statistical Analysis

The data were presented as mean ± standard deviation of triplet experiments, which were analyzed using Student’s t-test, and values of *p* < 0.05 were considered statistically significant.

## 3. Results and Discussion

### 3.1. Expression, Purification, and Characterization of MYR-coreSA Fusion Protein

**Figure 3:**
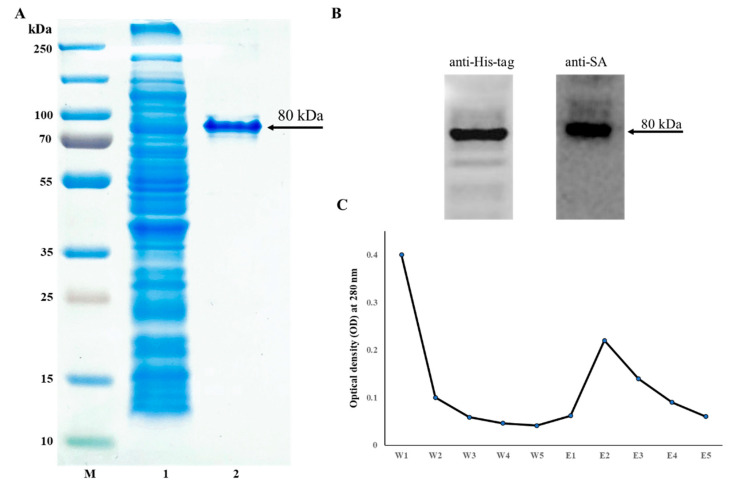
(**A**) SDS-PAGE imaged as (M) protein ladder, (1) crude bacterial lysate, (2) purified MYR-coreSA fusion protein; (**B**) Western blot of purified MYR-coreSA fusion protein against anti-His-tag and anti-SA; (**C**) elution profile of MYR-coreSA fusion protein using five washes and five elutions.

The pMT002 plasmid was constructed by cloning MYR and coreSA in between EcoRV/BamHI and EcoRI/XhoI of the pET-30a(+) vector, respectively. As shown in Figure 2B, the gene sequences encoding MYR and SA from pMT002 cloned by PCR were verified by DNA gel electrophoresis with ~1.6 kb for MYR and ~0.4 kb for SA. The estimated fusion protein size was calculated as ~80 kDa (15 + 60 + 5) from the coreSA being 387 bp (~15 kDa), MYR being 1623 bp (~60 kDa), and extras (6xHis, S tag, EK site, and other cloning sites of a vector) being 135 bp (~5 kDa). The SDS-PAGE in Figure 3A shows the crude lysate (Lane 2) and a single band of purified fusion protein around 80 kDa (Lane 3). The elution profile at OD_280_ was plotted, which shows the peak at the E2 fraction when most of the fusion protein was eluted (Figure 3C). The elutions were concentrated using a protein concentrator and quantified through BCA assay, and the concentration of purified protein was calculated as 268 ± 9 µg/mL. Moreover, it was further characterized by Western blot against monoclonal antibody streptavidin and monoclonal His-tag antibody, and the clear band in Figure 3B confirms the MYR-coreSA fusion protein.

**Figure 4:**
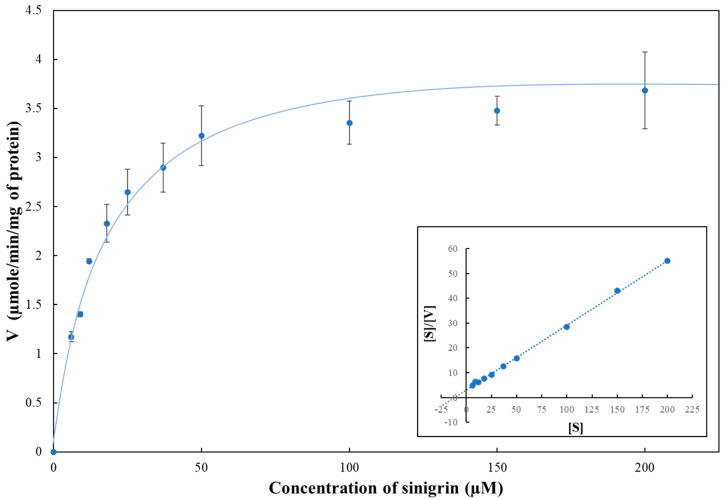
Enzyme activity assay of MYR-coreSA fusion protein using sinigrin as substrate at 37 °C and pH 7. Kinetic plot of specific activity V (µmol/min/mg protein) versus substrate concentration (µM). The Hanes plot (shown as inset) was employed to estimate *K_m_* and *V_max_*.

The enzyme activity of recombinant MYR-coreSA protein was measured using sinigrin as substrate (concentration ranging from 0 to 200 µM). The kinetic analysis was performed by plotting specific activity (µmol/min/mg protein) with respect to sinigrin concentration used, as shown in Figure 4. Furthermore, the Hanes plot (shown in the inset of Figure 4) was used to determine *V_max_* = 3.83 ± 0.4 µmol/min/mg protein and *K_m_* = 12.67 ± 2.15 µM, which are close to the values reported previously [24]. This also suggests that the chimeric protein of myrosinase with core streptavidin has no effect on its stability and functionality.

### 3.2. Biotinylation of Cell Membrane Surface

**Figure 5:**
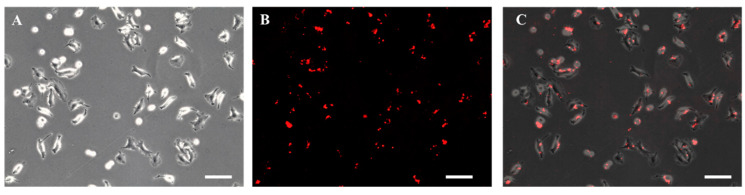
Photomicrographic images of A549 cells treated with 0.02 mg/mL biotin-X DHPE followed by the treatment of streptavidin labeled with PE-Cy5.5 for 24 h: (**A**) phase-contrast, (**B**) fluorescence, and (**C**) overlay image (scale bar = 100 µm).

The A549 cells were incubated for 24 h in culture media supplemented with 0.02 mg/mL of biotin-X DHPE for biotinylation. As shown in Figure 5A, the biotinylated A549 cells were able to proliferate without any change in morphology and obvious toxicity. It has been reported using biotin–lipid-containing culture medium to successfully biotinylate the surface of Vero cells [25] and mesenchymal stem cells [26]. Figure 5B also illustrates biotinylation of A549 cells was confirmed by incubating for 24 h with streptavidin labeled with PE-Cy5.5 (excitation/emission wavelength = 488/694 nm).

### 3.3. Anticancer Activity of Sinigrin on Myrosinase-Tethered A549 Cells

**Figure 6:**
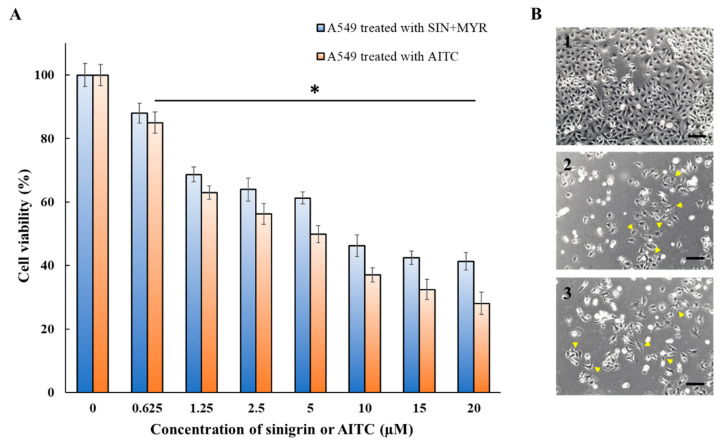
(**A**) Cell viability of biotinylated A549 cells after treatment with AITC, SIN+MYR for 48 h, as measured with the MTT assay; (**B**) phase-contrast images of biotinylated A549 cells after 48 h: (**B1**) control (i.e., DMSO treated); (**B2**) AITC treated (20 µM); (**B3**) SIN+MYR treated. Yellow arrowheads indicate cell morphological changes probably due to apoptosis (scale bar = 100 µm). * Denotes *p* < 0.05 in comparison to the control (i.e., 0 μM).

To assess the anticancer activity of the sinigrin–myrosinase system, A549 cells were cultured up to 40% confluency, biotinylated, and decorated with MYR-coreSA. The cells were then treated with different concentrations of sinigrin and AITC. After 48 h, cell viabilities were estimated with respect to control (cells treated with 1% DMSO only) using the MTT assay. Figure 6A shows that the viability of biotinylated A549 tethered with MYR-coreSA and treated with 20 µM of sinigrin decreased to ~40% compared to the control group. For biotinylated A549 cells treated directly with 20 µM of AITC (dissolved in 1% DMSO), cell viability decreased to ~30% compared to the control group. The IC_50_ for biotinylated A549 cells tethered with MYR-coreSA and treated with sinigrin was estimated to be ~8 µM and for biotinylated A549 cells treated directly with AITC was estimated to be ~5 µM, which are very close to previously reported study [27]. The phase-contrast images were also taken after 48 h cultivation for the three groups. As shown in Figure 6(B1), biotinylated A549 cells treated with DMSO show no visible changes in morphology and no negative effect on cell proliferation. However, biotinylated A549 cells treated with 20 µM of AITC directly (Figure 6(B2)) and biotinylated A549 cells tethered with MYR-coreSA and treated with 20 µM of sinigrin (SIN+MYR; Figure 6(B3)) show significant cell death and morphological change. As illustrated, the cells (with arrowheads) were shrunk and probably underwent apoptosis, which is consistent with the previously reported morphology of apoptotic cells [28,29,30]. Furthermore, biotinylated A549 cells were treated with 20 µM of sinigrin only and did not exhibit cell toxicity and morphological changes (data not shown), and we already reported biotinylation of A549 cells and streptavidin has no toxic effect on the cells [31]. Hence, the anticancer effectiveness was due to the conversion of sinigrin into AITC in the presence of myrosinase decorated on the outer surface of A549 cells. However, the anticancer activity was slightly higher when AITC was administered directly compared to AITC derived from sinigrin hydrolysis via myrosinase tethered on the cell surface (i.e., SIN+MYR). It is surmised that the enzymatic conversion of sinigrin into AITC was not 100%.

### 3.4. AITC-Induced Cell Apoptosis

**Figure 7:**
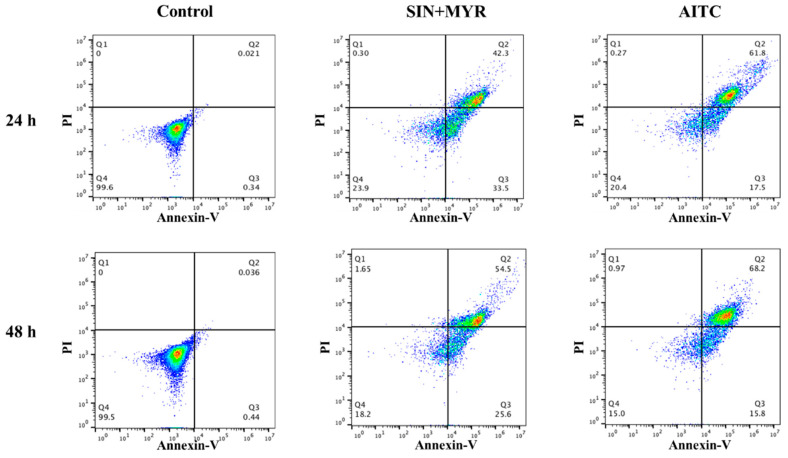
Flow cytometry analysis of A549 cells labeled with Annexin-V and PI for control, SIN+MYR, and AITC groups. The top panel shows the bivariate density plots after 24 h, and the bottom panel shows bivariate density plots after 48 h.

To examine the effect of sinigrin on arresting the growth of myrosinase-tethered A549 cells through apoptosis, cells were cultured and treated separately with SIN+MYR and AITC and then detected by the level of Annexin-V and Caspase-3/7 expressed. Figure 7 shows the flow cytometric bivariate density plots (Annexin-V vs. PI) for the control, SIN+MYR, and AITC groups after 24 h and 48 h. The percentage of necrotic cells (detected by PI and shown in Q2 zone) for myrosinase-bound cells treated with sinigrin (i.e., SIN+MYR) for 24 h and 48 h was 42.3% and 54.5%, respectively. For the AITC-treated group, relatively higher 61.8% and 68.2% of cell necrosis were observed after 24 h and 48 h, respectively. After 48 h, 54.5% of dead cells treated with SIN+MYR and 68.2% of dead cells treated with AITC are consistent with the percentages of cell viability (~60% and ~70%, respectively) determined by the MTT assay (as shown in Figure 6). The percentage of apoptotic cells (detected by Annexin-V and shown in Q3 zone) for myrosinase-bound cells treated with sinigrin (i.e., SIN+MYR) for 24 h and 48 h was 23.9% and 18.2%, respectively. For the AITC-treated group, relatively lower 17.5% and 15.8% of cell apoptosis were observed after 24 h and 48 h, respectively. Both groups (SIN+MYR and AITC) showed the same trends: the treated cells underwent apoptosis first and then turned into necrosis. As shown in Figure 7, the percentage of viable cells of the SIN+MYR group reduced from 23.9% to 18.2% after 48 h treatment, and the percentage of apoptotic cells reduced from 33.5% to 25.6%, yet the percentage of necrotic cells increased from 42.3% to 54.5%. This pattern of the treated cells undertaken apoptosis first and then turned into necrosis was also observed for the cells treated with AITC only. Our flow cytometric results showed similar trends to previously reported studies of AITC on different cancer models, i.e., the cell death was increased in a time-dependent manner [18,27,32,33].

**Figure 8:**
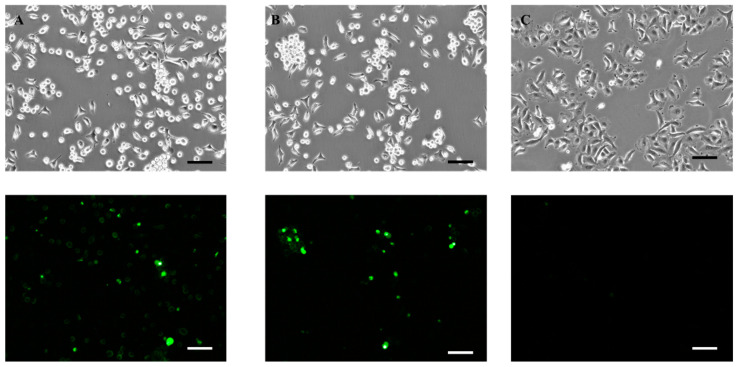
Phase-contrast images of A549 cells treated with (**A**) AITC, (**B**) SIN+MYR, and (**C**) DMSO (top panel), and fluorescence images were taken with the Caspase-3/7 green detection reagent (bottom panel). The fluorescent cells confirm the Caspase-3/7 activation, and no fluorescence was detected in the cells treated with DMSO as the control group (scale bar = 100 µm).

The Caspase-3/7 green detection reagent has DEVD (four amino acid) peptide conjugated with green dye (excitation/emission: 503/530 nm) with the ability to bind with nucleic acid, but the dye is nonfluorescent until it cleaves from the DEVD peptide and binds to DNA. The caspases are primary mediators of apoptosis with Caspase-2, -8, -9, and -10 as initiator and Caspase-3, -6, and -7 as effector caspases [34]. Caspase-3 is an important effector. When it is activated in apoptotic cells, the DEVD peptide is cleaved, which enables the green dye to bind the DNA and bright fluorescence is produced. After 24 h treatment of SIN+MYR and AITC, A549 cells were incubated with the Caspase-3/7 green detection reagent, which showed green fluorescent under the fluorescent microscope, confirming the activation of Caspase-3/7 and, hence, apoptosis in the treated cells (Figure 8).

Currently, several chemotherapy drugs are used for the treatment of cancer, which is highly toxic and comes with severe side effects such as anemia, hair loss, nausea, etc. Therefore, alternative therapeutic approaches are needed that target cancer cells specifically and have low toxicity to healthy cells, hence reducing side effects. One such approach is the development of chemopreventive drugs from natural resources (e.g., plant derived), as they are considered safer with fewer side effects [35]. AITC, a naturally occurring isothiocyanate, is abundant in cruciferous vegetables, and its chemopreventive properties have been reported in the literature. However, the reported concentration of AITC used for the eradication of cells varies from cell line to cell line. For instance, the maximum reported AITC concentration was 40 µM for cisplatin-resistant oral cancer cells [20], 92.5 µM for bladder cancer [18], and 10 µM for lung cancer cells [27]. Nevertheless, in the current study, 20 µM of AITC and sinigrin was determined to achieve significant cell death after 48 h treatment.

AITC is volatile and stored in its precursor sinigrin, which is more stable. Since sinigrin is not a bioactive compound and converts to AITC with the help of an enzyme myrosinase, it can be used as a nontoxic prodrug for anticancer treatment. Previous studies mostly reported the direct effect of AITC on cancer cells both in vivo [11,13,36] and in vitro [14,33,37]. Though, rich mustard seed powder was used in some studies in which AITC was stored stably as sinigrin. With the addition of water, the endogenous myrosinase hydrolyzed the sinigrin and caused apoptosis [36]. However, in this study, we used pure sinigrin, which produces AITC when it is contacted with myrosinase tethered on the surface of cancer cells. Our approach is more targeted and safer, as sinigrin has no toxic effect on cells. Some in vitro studies have shown the delivery of AITC using nanoparticles [38,39,40]; however, such an approach poses side effects because of potential leaking out of AITC before nanocarriers arrive at targeted sites for in vivo delivery. To circumvent this detrimental AITC release causing collateral damage to healthy normal cells, in situ production of AITC is desirable and therefore administration of sinigrin as a prodrug is safe, as it is biologically inactive until catalyzed into AITC when encountering myrosinase.

## 4. Conclusions

Myrosinase along with core streptavidin can be overexpressed in bacteria and purified using immobilized metal affinity chromatography to obtain MYR-coreSA chimeric protein, which shows a single clear band on SDS-PAGE and Western blot. It also has stable activity measured with enzyme kinetic assay using sinigrin as substrate. We have successfully biotinylated A549 cells and tethered them with myrosinase through streptavidin–biotin binding. The myrosinase-tethered A549 cells were treated with sinigrin to induce apoptosis, thereby resulting in significant cell death after 48 h treatment. Our study implies that sinigrin can effectively be used as a prodrug to induce apoptosis and ensuing cell death of myrosinase-expressed lung cancer cells. Such gene-directed myrosinase sinigrin therapy has the potential to produce AITC in situ to eradicate carcinomas without collateral damage.

## Figures and Tables

**Table 1:** Primers used for both inserts.

**Myrosinase (MYR)**	
**Forward primer**	5′-GCCATGGATATCATGAAGCTTCTTATGCTCGCCTTTG-3′
**Reverse primer**	5′-GAATTCGGATCCTGCATCTGCAAGACTCTTCCGATC-3′
**Core Streptavidin (coreSA)**	
**Forward primer**	5′-AGATCCGAATTCGGTGCTGCTGAAGCAGGT-3′
**Reverse primer**	5′-ATTATACTCGAGGGAGGCGGCGGACGGCTT-3′

## Data Availability

Not applicable.
